# Toxin-Based Targeted Therapy for Malignant Brain Tumors

**DOI:** 10.1155/2012/480429

**Published:** 2012-02-09

**Authors:** Vidyalakshmi Chandramohan, John H. Sampson, Ira Pastan, Darell D. Bigner

**Affiliations:** ^1^Department of Pathology, Duke University Medical Center, Durham, NC 27710, USA; ^2^Department of Surgery, Duke University Medical Center, Durham, NC 27710, USA; ^3^Laboratory of Molecular Biology, Center for Cancer Research, National Cancer Institute, National Institutes of Health, Bethesda, MD 20892, USA

## Abstract

Despite advances in conventional treatment modalities for malignant brain tumors—surgery, radiotherapy, and chemotherapy—the prognosis for patients with high-grade astrocytic tumor remains dismal. The highly heterogeneous and diffuse nature of astrocytic tumors calls for the development of novel therapies. Advances in genomic and proteomic research indicate that treatment of brain tumor patients can be increasingly personalized according to the characteristics of the targeted tumor and its environment. Consequently, during the last two decades, a novel class of investigative drug candidates for the treatment of central nervous system neoplasia has emerged: recombinant fusion protein conjugates armed with cytotoxic agents targeting tumor-specific antigens. The clinical applicability of the tumor-antigen-directed cytotoxic proteins as a safe and viable therapy for brain tumors is being investigated. Thus far, results from ongoing clinical trials are encouraging, as disease stabilization and patient survival prolongation have been observed in at least 109 cases. This paper summarizes the major findings pertaining to treatment with the different antiglioma cytotoxins at the preclinical and clinical stages.

## 1. Introduction

National Cancer Institute statistics predicted 22,020 new cases of malignant brain tumor and 13,140 brain tumor-related deaths to be recorded in the United States in 2010. Glioblastoma multiforme (GBM) is the most common primary malignant brain tumor of the central nervous system [1]. Hallmarks of GBM include uncontrolled cellular proliferation, diffuse infiltration, necrosis, angiogenesis, resistance to apoptosis, and genomic instability. Further, GBM exhibits considerable intratumoral heterogeneity, both at the cellular level and at the molecular level. The prevailing treatment options for GBM include surgery, radiation therapy, and chemotherapy [2–4]. The median survival for GBM patients with the standard multimodal therapy, including maximum safe surgical resection, radiotherapy, and concomitant chemotherapy with temozolomide (TMZ), is 14.6 months from the time of diagnosis [5]. Progression-free survival for recurrent GBM with currently available salvage therapies is less than 24 weeks, and most patients develop progressive disease within 8 to 10 months and die from refractory tumor soon thereafter [6]. Despite these efforts to overcome GBM, the nonspecific nature of conventional therapy for brain tumors often results in damage to the surrounding normal brain and systemic tissue [7, 8]. As a result, there is an urgent need for the development of therapeutics designed to specifically target tumor cells while preserving the adjacent normal tissue.

Current understanding of molecular abnormalities associated with glioma oncogenesis has identified distinct biological features common to glioma but atypical in normal brain tissue [9, 10]. Differential expression of tumor-specific proteins warrants selective targeting of tumor cells, with low toxicity to the surrounding normal tissue. These proteins are often described as tumor-associated antigens (TAAs) [11]. A majority of the human tumor antigens are either overexpressed normal gene products or those derived from mutations in somatic genes. Hence, TAAs are not strictly tumor specific. However, tumors often express these antigens at higher levels than do normal tissues (often up to 10,000-fold), and the accessibility of antigens on tumors may therefore be greater than in normal tissue [12].

## 2. Toxin-Based Therapeutics for Brain Tumor Treatment

The concept of targeting cancer cells using an antibody-toxin conjugate was investigated in 1970 by Moolten and Cooperband [13]. As a proof of concept, they explored the idea of employing an antibody-toxin conjugate against viral antigens overexpressed on the surface of monkey kidney cells. Their study successfully demonstrated that antibody-toxin fusion proteins could be utilized for targeting neoplastic cells. The development of monoclonal antibodies (mAbs), and specifically, the identification of tumor-antigen-specific mAbs, spearheaded the exploitation of immunotoxins to kill cancer cells. Immunotoxins, otherwise known as cytotoxins, are recombinant molecules that specifically bind to antigens overexpressed on the surface of a cancer cell [14]. These recombinant proteins consist of a specific antibody or ligand coupled to a toxin protein. The toxins used in the construction of cytotoxins are natural byproducts of plants, bacteria, and fungi that inactivate eukaryotic protein synthesis.

The most commonly employed toxins in the construction of immunotoxins include the bacterial toxins *Pseudomonas aeruginosa* exotoxin A (PE) and diphtheria toxin (DT) and the plant toxin ricin. All three toxins, PE, DT, and ricin, are synthesized as single-polypeptide chains consisting of functionally distinct domains, and these toxins all belong to the class of A-B toxins, which require cellular uptake through receptor-mediated endocytosis for activity [15, 16]. The B subunit of these proteins encodes a receptor-binding domain (B subunit) linked to the A subunit with cytotoxic activity. Despite their diversity in size, subunit composition, cell specificity, and enzymatic activity, these toxins share a similar function—protein synthesis inhibition—either by inhibiting elongation factor 2 (EF2) or 60S ribosome. A single molecule of toxin can irreversibly inactivate 300 ribosomes in 35 min and is sufficient to kill a cancer cell [16–19].

### 2.1. Pseudomonas Exotoxin A

*Pseudomonas *exotoxin A is expressed as a single 638-amino-acid polypeptide by the Gram-negative bacterium *Pseudomonas aeruginosa.* Upon synthesis, a 25-amino-acid segment is clipped off the N-terminus of the proprotein, and a 613-amino-acid mature toxin is secreted [16]. The 66 kDa mature toxin comprises three major functional domains [20, 21]. The N-terminal domain I, which is subdivided into domains Ia (residues 1–252) and Ib (365–404), is the receptor-binding domain, and it targets the low-density, lipoprotein-receptor-related protein (LRP1), or the closely related variant LRP1B expressed in the plasma membrane of mammalian cells for subsequent cellular internalization by receptor-mediated endocytosis [22, 23]. Domain II is composed of residues 253–364 and is involved in toxin translocation and intracellular trafficking. The remaining C-terminal residues (405–613), along with a portion of domain Ib (residues 395–404), make up the catalytically active domain, and they catalyze the adenosine-diphosphate- (ADP-) ribosylation and inactivation of EF2, which leads to inhibition of protein synthesis followed by cell death [24]. The earliest PE-based therapeutics application was generated by chemical coupling of the full-length PE to intact antibodies [25]. Elucidation of the structure and function of PE led to the development of recombinant immunotoxins, wherein the receptor binding domain of PE was substituted with an antibody, the Fv portion of an antibody, a growth factor, or a cytokine, to generate a higher binding affinity to cancer cells, through chemical conjugation or recombinant DNA technology [14].

### 2.2. Diphtheria Toxin

Diphtheria toxin is secreted by the Gram-positive bacterium *Corynebacterium diphtheriae* as a single polypeptide chain of 535 amino acids [26]. The functional DT is composed of two major domains: amino terminal domain A (1–193 amino acids) carries the active site for ADP-ribosylation of EF2, and carboxyl terminal domain B (194–535 amino acids) promotes binding of toxin to cells and the entry of domain A into the cytosolic compartment. The human heparin-binding epidermal growth factor-like precursor (HB-EGF) acts as the receptor for DT on the plasma membrane of human cells [27]. Once internalized by receptor-mediated endocytosis, an arginine-rich segment connecting the A and B domains is readily cleaved by trypsin-like enzymes to yield active fragments A and B  [28]. In the acidic conditions of the late endosome, domain A is translocated into the cytosol [29], where it catalyzes the transfer of the ADP-ribose moiety of nicotinamide adenine dinucleotide (NAD+) to a highly conserved diphthamide residue (modified histidine) on the EF2 polypeptide chain, thereby inactivating it [30–32]. Modified EF2 can no longer make new protein, and the cell dies via apoptosis. Recombinant-DT-based immunotoxins are constructed by mutating or replacing the carboxyl terminal cell binding domain and fusing the engineered toxin with a ligand to a cancer cell surface receptor or the Fv fragment of an antibody.

### 2.3. Ricin

The plant toxin ricin, extracted from the seeds of *Ricinus communis*, belongs to a group of toxins called ribosome-inactivating proteins (RIPs) Type II [12]. Ricin is synthesized as a precursor protein of 565 amino acids, consisting of a 24-amino-acid N-terminal signal sequence followed by the A chain (267 amino acids), which in turn is attached to the B chain (262 amino acids) by a 12-amino-acid linker [33]. During biosynthesis, the signal sequence is cotranslationally removed from preproricin to generate proricin, and the 12-amino-acid linker is cleaved posttranslationally to yield the mature protein held together by a disulfide bond. The toxic action of ricin is associated with the A chain, and the B chain functions as a carrier moiety which binds the toxin to the galactose-containing receptors on the cell surface [34, 35]. Once the A chain reaches the cytosol of the target cell, it enzymatically attacks the 28S rRNA in the 60S ribosomal subunit and disrupts protein synthesis [15, 36, 37]. Immunotoxins are generated by chemical cross-linking of the intact ricin or the recombinant ricin A chain to antibodies directed to antigens overexpressed on the tumor cell surface.

## 3. Development and Evaluation of Immunotoxins/Cytotoxins Targeting Brain Tumor Antigens in the Preclinical and Clinical Settings

In 1905, Paul Ehrlich proposed the idea of using antibodies to deliver chemotherapeutic agents. Since then, the utility of antibodies to specifically diagnose, localize, and treat human tumors has been actively pursued. A tumor-specific antigen expressed only by the tumor cell and not by normal cells is hard to discover. However, many molecular targets that are expressed in much higher concentrations on glioma cells, compared to those on normal neural tissues, have been identified, and mAbs against these glioma-associated antigens (GAAs) have been developed [50]. Several of these antigens and their therapeutic utilities are discussed below and summarized in Table 1.

### 3.1. Transferrin Receptor

The transferrin receptor (TR) is a transmembrane glycoprotein which mediates the cellular uptake of iron from the plasma glycoprotein transferrin [51]. TR is expressed in all nucleated cells of the body, with expression levels corresponding to their iron requirements and proliferative status. Due to the increased growth and rapid proliferation capacity of cancer cells, overexpression of TR has been observed in several cancer types [52]. The use of an anti-TR mAb chemically coupled to the subunits of DT or ricin to target tumor was first investigated by Trowbridge and Domingo [53]. Since then, several anti-human TR mAb ricin-based immunotoxins have been successfully tested *in vitro* for the treatment of malignant brain tumors [54, 55]. The *in vitro* and *in vivo* efficacy of two such targeted protein toxins was studied: (1) Tf-CRM107, a conjugate of human TR and full-length DT with two point mutations in the B chain with reduced toxin binding (CRM107) [56], and (2) 454A12-rRA, a conjugate of a mAb (454A12) to the human TR and recombinant ricin A chain (rRA), against TR-expressing brain tumors. Both compounds exhibited significant killing of human glioblastoma cell lines *in vitro* and *in vivo*. However, Tf-CRM107 was more potent than 454A12 [57–59].

 Leptomeningeal neoplasia occurs in 5%–20% of all cancer patients and results in a very poor prognosis, with a median survival of only a few months [60]. A phase I study was conducted to identify the toxicity, pharmacokinetics, and antitumor activity with the 454A12-rRA immunotoxin in eight patients with leptomeningeal spread of systemic neoplasia (six patients with breast carcinomas, one patient with melanoma, and one patient with leukemia) [40]. In four of the eight patients, the lumbar cerebrospinal fluid (CSF) tumor cell counts dropped by more than 50% within 5–7 days after the intraventricular delivery of 454A12-rRA; however, no patient had the CSF cleared of tumor, and evidence of tumor progression was demonstrated in seven of the eight patients after treatment.

 The toxicity, maximum-tolerated dose (MTD), and efficacy of Tf-CRM107 was evaluated in a phase I single-center, dose-escalating, single-arm trial, in patients with malignant brain tumors refractory to conventional therapy [38]. The Tf-CRM107 was directly delivered to tumor and surrounding infiltrated brain by high-flow interstitial microinfusion. Eighteen patients were enrolled for the trial, and fifteen patients were evaluated for radiographic evidence of tumor regression. Following Tf-CRM107 infusion, a ≥50% decrease in tumor volume occurred in nine of the 15 patients (60%) evaluated [38]. The treated tumors showed complete responses (CRs) in two of the patients, with one of these two patients showing no evidence of tumor for 23 months after a single infusion of Tf-CRM107 into a progressing recurrent GBM [38]. The results demonstrated that Tf-CRM107, delivered via convection-enhanced drug delivery (CED), can elicit antitumor activity without severe neurologic or systemic toxicity in malignant brain tumor patients.

 A phase II multicenter trial by intratumoral CED infusion of Tf-CRM107 for patients with recurrent GBM or anaplastic astrocytoma (AA) was launched. Patients then received Tf-CRM107 infusions (0.67 *μ*g/mL, up to 0.40 mL/h), for 4-5 days until a volume of 40 mL was delivered [39]. Four to 10 weeks after initial infusion the patients were readmitted to receive a second treatment. Efficacy response for the treated tumors was categorized as complete response, partial response (PR), stable disease, or progressive disease. Of 34 evaluable patients, the Tf-CRM107 treatment resulted in CRs in a total of five patients and PRs in seven patients (35%, *P* < 0.0001). The median survival time was 37 weeks, 13/44 patients (30%) survived beyond 12 months from the time of the first infusion, and one patient survived 1144 days. In 8 of the 44 12-month survivors (14%), Tf-CRM107 infusions caused symptomatic progressive cerebral edema, which responded to medical management. Seizures were seen in 3 of these 44 patients (7%) and were treated by anticonvulsant therapy [39]. The phase II study indicated that tumor response can be obtained in a significant percentage of recurrent or refractory GBM or AA patients, and the magnetic resonance imaging (MRI) tumor response correlated with a more favorable survival. A phase III clinical trial with Tf-CRM107 for patients with nonresectable, progressive, or recurrent GBM who had failed conventional therapy was initiated in 2004. But the study was discontinued in 2007 because of a failure to demonstrate a positive therapeutic response. Future trials could focus on increasing the treatment efficacy of Tf-CRM107 by combining this agent with other toxin conjugates targeted to different GAAs or by employing a genetically engineered Tf with decreased iron release rate and increased cellular association and cytotoxicity [61].

### 3.2. Interleukin-4 Receptor

Interleukin-4 (IL-4) is a pleiotropic cytokine produced by TH2-type CD4+ T cells, basophils, and mast cells, in response to receptor activation. IL-4 regulates the differentiation of antigen-stimulated naive T cells and the specificity of immunoglobulin class switching by B cells [62]. IL-4 functions by signaling through its receptor. IL-4 receptors are expressed in several cell types, including hematopoietic, endothelial, muscular, and neuronal cells, and are usually expressed at low levels ranging from 100 to 5000 receptors per cell [62]. Two types of IL-4 receptors have been identified so far. Type I receptors consist of a 140-kDa protein, IL-4R*α* (also known as IL-4R*β*), and the *γ* common (*γ*_c_) chain [63, 64]. Type II receptors are composed of IL-4R*α* and IL-13R*α*1 (also known as IL-13R*α*′) chains [65, 66]. The type II receptor is expressed mainly in nonhematopoietic cells. Ligand-induced heterodimerization of IL-4R activates cytoplasmic tyrosine kinases (Janus kinase family-Jak), which in turn phosphorylates cellular substrates and initiates signaling cascades leading to cellular proliferation and gene expression [62].

Several human tumors are known to express IL-4R, including malignant melanoma, breast carcinoma, ovarian carcinoma, and renal cell carcinoma [67, 68]. A chimeric protein composed of human IL-4 and a full-length PE molecule with mutation in the cell binding domain of PE, hIL4-PE4E, was cytotoxic (with 50% inhibition of protein synthesis [IC_50_] occurring in the 12–120-pM range) to a wide range of human cancer cell lines [69, 70]. The high-affinity IL-4 receptors were also demonstrated to be present on human GBM, neuroblastoma, and glioma cells, and IL-4-PE4E was found to be highly cytotoxic (with IC_50_ in the range of 85–2000 pM) to the neurological tumor cells [71]. Attachment of the large PE molecule to the carboxyl terminus of hIL-4 decreased the affinity of the hIL-4-PE4E toxin by 15–20-fold compared to that of native hIL-4 [69]. To overcome this hurdle, a circularly permuted recombinant IL-4 was developed by connecting the IL-4 amino acids 38–129 via the linker GGNGG to amino acids 1–37, which were in turn fused to a redesigned PE containing amino acids 253–364 and 381–608, followed by a new endoplasmic reticulum retention sequence, KDEL [72, 73]. This new toxin, termed IL-4(38-37)-PE38KDEL, exhibited improved binding affinity, cytotoxicity (with IC_50_ in the 6–17-pM range), and *in vivo* antitumor activity against IL-4R-expressing cancers [72, 73]. Further studies demonstrated the expression of IL-4 receptor in malignant brain tumor samples, which were highly sensitive to IL-4(38-37)-PE38KDEL treatment, whereas it was less toxic to hematopoietic and normal brain cells [74, 75]. Intratumoral administration of IL-4(38-37)-PE38KDEL into U251 glioblastoma flank tumors in nude mice induced complete regression of tumors in all of the animals without any toxicity [76]. Preclinical investigation in rats demonstrated that significant cytotoxic activity against malignant astrocytic tumors could be achieved by intrathecal administration of IL-4(38-37)-PE38KDEL, with no toxicity to normal cells [75].

Based on the preclinical studies, a phase I clinical trial was initiated to determine the safety and tolerability of IL-4(38-37)-PE38KDEL (NBI-3001) in recurrent human malignant glioma when injected intratumorally by CED by 2-3 catheters [41]. No histological evidence of neurotoxicity to normal brain was identified in any patient. Six of nine patients showed glioma necrosis, as evidenced by decreased enhancement on MRI. Of six patients, one patient remained disease free for >18 months after the procedure [41]. A multicenter open-label dose-escalation trial was launched to determine the MTD, volume, and safety of IL-4(37-38)-PE38KDEL when injected stereotactically [42, 43]. A total of 31 patients, 25 GBM and 6 AA, were assigned to one of four dose groups in a dose-escalation fashion: 6 *μ*g/mL in 40 mL, 9 *μ*g/mL in 40 mL, 15 *μ*g/mL in 40 mL, or 9 *μ*g/mL in 100 mL of IL-4(37-38)-PE38KDEL administered intratumorally via stereotactically placed catheters [42, 43]. No drug-related systemic toxicity was evident in any treated patients, and treatment-related adverse effects were limited to the central nervous system (CNS). Drug-related grade 3 or 4 CNS toxicity was observed in a total of 39% patients in all groups, and in 22% of patients at the MTD dose of 6 *μ*g/mL in 40 mL. The overall median survival for the whole group was 8.2 months, with a median survival of 5.8 months for the GBM patients. Six-month survival was 52% and 48%, respectively. MRI of the brain showed areas of decreased signal intensity within the tumor consistent with tumor necrosis following treatment in many patients [42, 43].

A multicenter, randomized, open-label phase II study with IL-4(38-37)-PE38KDEL administered to recurrent GBM patients was conducted to evaluate the efficacy, safety, and tolerability of the toxin after continuous intratumoral infusion of the toxin at total doses of up to 90 *μ*g followed by surgical resection of the tumor [44]. A total of 30 adult patients with unilateral, unifocal tumor with a volume of 5–80 mL and a Karnofsky performance score (KPS) of ≥70 were enrolled (ClinicalTrial.gov identifier: NCT00014677). The results of the study have not been published. Currently, there are no phase III protocols involving IL-4(38-37)-PE38KDEL.

### 3.3. Interleukin-13 Receptor

Interleukin-13 (IL-13) is a 12-kDa cytokine that is expressed by T cells, mast cells, and Epstein-Barr-virus- (EBV-) transformed B cells. IL-13 protein has 30% amino acid sequence homology with the IL-4 protein, and, hence, these cytokines share a variety of functions [65]. Functions of IL-13 include upregulation of major histocompatibility complex (MHC) class II and low-affinity IgE receptor expression on monocytes, induction of IgE and IgG4 class switching in B cells, and upregulation of vascular cell adhesion molecule-1 (VCAM-1) expression on endothelial cells [65]. Two receptors for IL-13 have been identified: IL-13R*α*2, a membrane-bound protein with a high affinity for IL-13, and IL-13R*α*1, a low-affinity IL-13 binding chain. IL-13R*α*1 upon interaction with the IL-4R*α* binds IL-13 with high affinity and activates an intracellular signaling pathway in tumor, endothelial, fibroblast, and immune cells [77]. IL-13R*α*2 binds IL-13 in an IL-4-independent manner [78].

The IL-13R*α*2 is expressed in many human tumors, including GBM, acquired immunodeficiency syndrome-associated Kaposi sarcoma, squamous cell carcinoma of the head and neck, pediatric brain tumors, and medulloblastoma [78–84]. Human glioma cells overexpressing IL-13R were highly sensitive (IC_50_ at <20 pM) to a chimeric toxin composed of hIL-13 and a mutated version of PE, PE38QQR-IL-13-PE38QQR, which has lysines 590 and 606 replaced with glutamines and lysine 613 with arginine [80]. A second fusion protein composed of hIL-13 and the first 389 amino acids of DT, DT_390_IL13, exhibited significant cytotoxicity against established and primary GBM cell lines and completely regressed GBM tumors in nude mice [85, 86]. A recombinant immunotoxin, anti-IL-13R*α*2(scFv)-PE38, was also constructed by fusing human single-chain variable fragments (scFvs), isolated from a phage library, against IL-13R*α*2 with PE. The antitumor activity against GBM tumors with anti-IL-13R*α*2(scFv)-PE38 was not higher than that with IL-13-PE38 [87].

 Encouraging preclinical results have led to the initiation of several phase I/II clinical trials in recurrent malignant glioma patients with IL13-PE38QQR (also known as Cintredekin Besudotox; CB; NeoPharm, Lake Bluff, IL). Intracerebral CED of IL-13-PE38QQR following tumor resection was evaluated in three phase I clinical trials. The aim was to assess the tolerability of different concentrations and infusion durations, tissue distribution, and methods for optimizing delivery of IL-13-PE38QQR [45]. A total of 51 patients (46 GBM, 3 AA, 1 anaplastic oligoastrocytoma, 1 anaplastic oligodendroglioma) were treated. All patients underwent tumor resection followed by intraparenchymal placement of one to three catheters in areas at risk for residual infiltrating tumors. IL-13-PE38QQR was then administered by CED at a fixed total infusion rate of 0.750 mL/h divided by the number of catheters for 96 h. An intratumoral IL-13-PE38QQR infusion preceding tumor resection was also performed in 18 of 51 patients to assess drug distribution. Intraparenchymal concentrations of 0.25 *μ*g/mL and 0.5 *μ*g/mL were well tolerated with infusion durations up to 6 days. The MTD for intraparenchymal concentration was 0.5 *μ*g/mL, and tumor necrosis was observed at this concentration. Postoperative catheter placement appears to be important for optimal drug distribution. IL-13-PE38QQR and procedure-related adverse events were primarily limited to the CNS. An overall median survival of 45.9 weeks was observed for the entire group posttreatment (*N* = 51). Median survival for GBM patients after treatment was 42.7 weeks (*N* = 46). The median survival was 55.6 weeks for GBM patients with two or more optimally positioned catheters (*N* = 24) and 37.4 weeks for GBM patients with fewer than two catheters optimally positioned (*N* = 19). Nine patients (17.6%) and seven patients (13.7%), all with GBM except one, had a prolonged progression-free survival beyond 1 and 2 years, respectively, with patient follow-up extending beyond 5 years [45].

 A phase III randomized evaluation of CED of IL13-PE38QQR compared to Gliadel wafer (GW; Rhone-Poulenc Rorer, Inc. (Paris, France), and Guilford Pharmaceuticals, Inc. (Baltimore, MD, USA)) with survival endpoint trial (called the PRECISE Trial), was conducted, and 296 patients were enrolled at 52 centers worldwide [46]. The aim of the study was to determine whether IL-13-PE38QQR therapy in recurrent GBM patients improved the overall survival duration, safety, and quality of life as opposed to GW treatment. Patients were randomized, 192 to receive IL-13-PE38QQR and 104 to receive GW. Of these 296 patients, a total of 276 patients had histopathologic confirmation of GBM. IL13-PE38QQR (0.5 *μ*g/mL; total flow rate of 0.75 mL/h) was administered over 96 h via 2–4 intraparenchymal catheters placed 2–7 days after resection in areas at greatest risk for infiltrating disease or in the vicinity of any residual, solid, contrast-enhancing disease. GWs (3.85%/7.7 mg carmustine per wafer; maximum 8 wafers) were placed immediately following tumor resection [46]. The total number of patients available for safety analysis with IL13-PE38QQR or GW (i.e., they received any study drug) and efficacy analysis was 269 and 188, respectively. Median survival for the GBM population was 36.4 weeks (9.1 months) for the IL-13-PE38QQR group and 35.3 weeks (8.8 months) for the GW group (*P* = 0.476). Median survival in the efficacy evaluable population was 45.3 weeks (11.3 months) for IL-13-PE38QQR and 39.8 weeks (10 months) for GW (*P* = 0.310) [46]. The safety and adverse events profiles were similar for both treatment groups, with the exception of pulmonary embolism, which was higher in the IL-13-PE38QQR group (8% versus 1%, *P* = 0.014). The PRECISE is the first and largest study using CED with an active comparator in recurrent GBM patients. Although IL-13-PE38QQR was well tolerated, there was no survival advantage of IL13-PE38QQR administered via CED compared with GW.

Since earlier studies described high levels of IL-13R*α*2 expression in a significant proportion of GBM samples, the IL-13R*α*2 levels were not examined in the GBM patients included in the current trial. A later study revealed IL-13R*α*2 to be expressed in only 44%–47% of GBM specimens, and even within individual positive tumors the distribution of IL-13R*α*2 was highly heterogeneous [88]. This study underscored the potential benefits of prescreening patients for IL-13R*α*2 expression prior to enrollment into IL13-PE38QQR-based targeted therapies. Further, the current trial did not include real-time imaging to ascertain if the requisite concentration of therapeutic agent was delivered to the tumor site. Hence, optimization of several parameters, including initial screening of patients for target antigen (IL-13R*α*2) expression, optimal catheter positioning, and real-time drug delivery imaging, should increase the success rate of future clinical trials.

### 3.4. Epidermal Growth Factor Receptor

The epidermal growth factor receptor (EGFR), a 170-kDa, transmembrane glycoprotein, is composed of three functional domains—an extracellular ligand-binding domain (ECD), an anchoring membrane-spanning region, and an intracellular catalytic domain that functions as a tyrosine kinase receptor. EGFR is stimulated by binding of its ligands, such as transforming growth-factor- (TGF-) *α* or EGF, to its ECD [89, 90]. Both EGF and TGF-*α* are small mitogenic proteins, each composed of 53 and 50 amino acid residues, respectively. TGF-*α* shares about 30% sequence identity with EGF, including the conservation of all six cysteines, which are involved in the formation of three intramolecular disulfide bonds [91]. The disulfide bonds are essential for their biological activity [92]. TGF-*α* competes with EGF for the same membrane-bound receptor sites [89]. Ligand binding induces receptor dimerization and activates a tyrosine-specific protein kinase activity [93] involved in controlling epithelial-cell growth and proliferation. Ultimately, the receptor-ligand complexes are internalized, and the EGFR signal is terminated.

 EGFR overexpression is frequently observed in a wide variety of human cancers, including breast [94], lung [95], head and neck [96], prostate [97], bladder [98], colorectal [99], and in ovarian carcinoma [100], as well as brain tumors [101, 102]. The ratio of EGFR expression in glioma versus normal control brain specimens has been shown to be as high as 300-fold [103]. *EGFR* is the most frequently amplified gene in GBM [104]. Correlating with the gene amplification, the protein is overexpressed in about 60%–90% of GBM cases. Even in the absence of gene amplification, protein overexpression has been observed in 12%–38% of GBM patients [105], which could be due to aberrant translational and posttranslational mechanisms. Preclinical studies have shown that EGFR activation, in addition to protecting cells from apoptosis, also induces several tumorigenic processes, including proliferation, angiogenesis, and metastasis [106].

Several chimeric toxins composed of TGF-*α* and PE, for example, TGF-*α*-PE38 and TGF-*α*-PE40, were constructed. These toxins were highly effective in retarding tumor growth and increasing the survival of athymic nude mice that were implanted with GBM or medulloblastoma xenografts [107, 108]. An anti-EGF receptor antibody (425.3), chemically conjugated to the whole PE molecule, was also investigated for the treatment of human gliomas. Treatment of intracerebral U87MG tumors in nude rats with 4 *μ*g of 425.3-PE increased symptom-free survival from 23 days to 40 days, with 2/9 rats surviving more than 90 days [109]. Further, a DT-based fusion protein was produced in conjunction with EGF, DAB_389_EGF, which exhibited significant cytotoxicity *in vitro* (with IC_50_ in the 0.4–50 pM range) and tumor regression *in vivo* against human GBM cells [47, 110].

The toxicity of TGF-*α*-PE38 (TP-38, IVAX) was assessed in athymic mice, athymic rats, and Rhesus macaques, and the MTD was determined to be 0.100 *μ*g, 0.666 *μ*g, and 2.0 *μ*g, respectively. Based on these studies, the estimated MTD for humans was 200 *μ*g [48]. Based on these preclinical data, a phase I study was initiated to define the MTD, dose-limiting toxicity (DLT), and efficacy of TP-38 delivered by CED for recurrent malignant glioma patients [49]. Twenty adult patients (17 GBM, 1 gliosarcoma (GSC), 1 metastatic spindle cell sarcoma, 1 anaplastic oligodendroglioma (AO)) with a KPS ≥60 were enrolled. TP-38 was infused over 50 h at a flow rate from each catheter of 0.4 mL/h for a total volume of 40 mL. Three escalating concentrations of TP-38, 25 ng/mL, 50 ng/mL, and 100 ng/mL, were selected for study. In the last eight patients, coinfusion of ^123^I-albumin was performed to monitor distribution within the brain [49]. Median survival for all patients after TP-38 treatment was 28 weeks and for those without radiographic evidence of residual disease, 33 weeks. Of the 15 patients treated with residual disease, two (13.3%) demonstrated radiographic responses, including one patient with GBM who had a nearly complete response and remains alive >260 weeks after therapy. Coinfusion of ^123^I-albumin demonstrated that high concentrations of the infusate could be delivered >4 cm from the catheter tip. However, only 3 of 16 (19%) catheters produced intraparenchymal infusate distribution, while the majority leaked infusate into the cerebrospinal fluid spaces. Due to adequate TP-38 delivery failure in a majority of the patients, the study was halted at a dose of 100 ng/mL, without reaching an MTD. Two DLTs were seen, and both were neurologic [49]. Intracerebral CED of TP-38 was well tolerated and produced some durable radiographic responses at doses <100 ng/mL. Future studies should concentrate on optimization of CED delivery technique and drug infusion parameters.

### 3.5. Mutant Epidermal Growth Factor Receptor Variant III

Several *EGFR* deletion mutants have been identified, the most common one being *EGFRvIII*, which is present in 20%–50% of GBMs with *EGFR* amplification [111]. The mutant *EGFRvIII* contains a deletion of exons 2–7 of the *EGFR* gene, which is characterized by an in-frame deletion of 801 base pairs of the coding region [112]. This deletion creates a novel glycine residue at the fusion junction at position 6, between amino acid residues 5 and 274, generating a tumor-specific protein sequence, that is, expressed specifically on tumor cells but not on normal tissues. EGFRvIII is a constitutively active receptor tyrosine kinase which is not further activated by EGFR ligands [113]. Like its wild-type counterpart, EGFRvIII is widely expressed in malignant gliomas [114] and carcinomas, including head and neck [115] and breast cancers [116]. Overexpression of EGFRvIII induces resistance in glioma cells to commonly used chemotherapeutic agents [117]. Hence, EGFRvIII is a desirable target for therapeutic intervention.

 The therapeutic efficacy of three anti-EGFRvIII-specific mAbs, L8A4, Y10, and H10, chemically coupled to PE35KDEL, was evaluated against a mutant EGFRvIII-expressing cell line. All three immunotoxins were cytotoxic, with an IC_50_ in the range of 15–50 pM [118]. An scFv specific for EGFRvIII, MR1 (mutant receptor), was isolated from an immunized phage library and was fused to an immunotoxin variant of PE, PE38KDEL. The cytotoxicity of MR1-PE38KDEL was tested against EGFRvIII-transfected malignant glioma cells, and the IC_50_ of MR1-PE38KDEL on these cells was in the range of 110–160 pM [119]. The toxicity and therapeutic efficacy of MR1-PE38KDEL was tested in an athymic rat model of neoplastic meningitis induced by intrathecal inoculation of an EGFRvIII-expressing human glioma [120]. A dose escalation study compared the survival of three equal doses of 1, 2, and 3 *μ*g of MR1-PE38KDEL with saline or 3 *μ*g of a control immunotoxin. All saline or control immunotoxin-treated animals died, with median survival of 7 and 10 days, respectively. There were 75% (1-*μ*g group) and 57% (2- or 3-*μ*g group) long-term survivors with MR1-PE38KDEL treatment. None of the MR1-PE38KDEL-treated groups reached median survival by the termination of the study at 53 days. Median survival was estimated to be >53 days. Compartmental therapy with three doses of 2 *μ*g of the MR-1 immunotoxin was effective in the treatment of EGFRvIII-expressing neoplastic meningitis, with no clinical or histopathological effects on nontumor-bearing animals [120].

**Figure 1:**
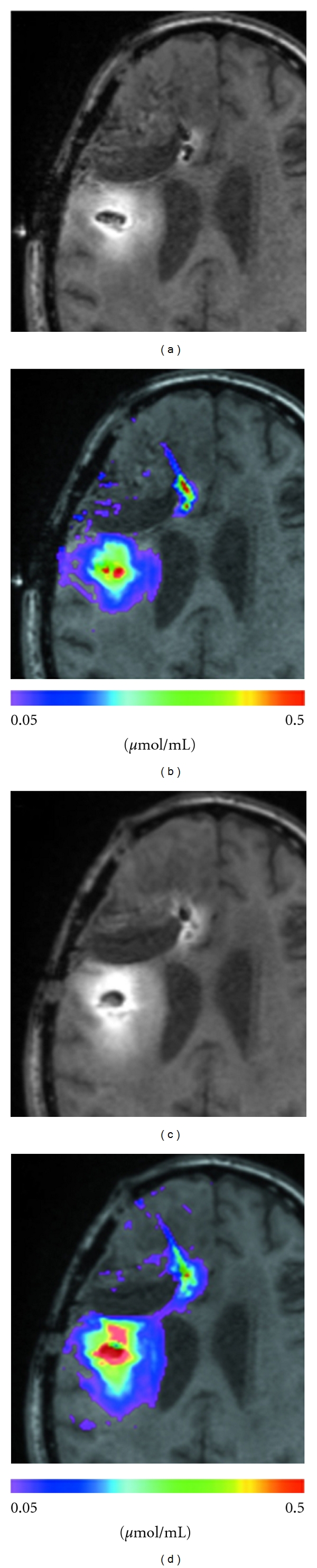
T1-weighted signal of MR1-1-PE38KDEL (a and c), compared with measured concentration profile of Gd-DTPA (b and d). (a) T1-weighted signal of MR1-1-PE38KDEL at 24 hours. (b) Concentration of Gd-DTPA at 24 hours. (c) T1-weighted signal of MR1-1-PE38KDEL at 72 hours. (d) Concentration of Gd-DTPA at 72 hours. Gd-DTPA, gadolinium-diethylene triamine pentaacetic acid. Reprinted from [122] with permission from Wolters Kluwer Health (license number 2747070143830).

An affinity-matured variant of MR1, termed MR1-1, with increased affinity to EGFRvIII, was generated for targeted glioma therapy [121]. The MR1-1 scFv was then fused to PE38, to generate MR1-1-PE38. As compared to the parental MR1-PE38, MR1-1-PE38 exhibited improved cytotoxic activity against the EGFRvIII-expressing NR6M cell line [121]. A phase I study to determine the MTD and DLT of MR1-1-PE38KDEL delivered intracerebrally by CED in patients with supratentorial malignant brain tumors is currently ongoing (ClinicalTrials.gov Identifier: NCT01009866). The study design includes infusion of MR1-1-PE38KDEL by CED using two intracerebral catheters. ^124^I-labeled albumin is coinfused with gadolinium to monitor radiographically the volume of drug distribution and leakage into the CSF space after drug infusion. A starting total drug dose of 0.5 *μ*g (1/20th of the MTD in rats) at a fixed flow rate of 0.5 mL/h is infused from each of the two catheters. A total of 96 mL of drug solution is delivered over 96 h. MR1-1-PE38KDEL dose escalation will be accomplished by increasing drug concentration while allowing flow rate and infusion volume to remain unchanged. Supratentorial malignant brain tumor patients with KPS >70 and expressing the EGFRvIII target antigen will be enrolled in this trial. The distribution of MR1-1-PE38KDEL at a concentration of 25 ng/mL by CED was monitored by coinfusion with the low molecular weight tracer gadolinium-diethylene triamine pentaacetic acid (Gd-DTPA) and ^124^I-labeled human serum albumin in a malignant glioma patient [122]. This study demonstrated that Gd-DTPA coinfusion is able to precisely demonstrate the distribution of MR1-1-PE38KDEL at the tumor site (Figure 1). Monitoring of MR1-1PE38KDEL distribution will aid in optimized drug dose deliverance at the tumor site thereby enhancing the therapeutic efficacy.

### 3.6. Glycoprotein Nonmetastatic Melanoma Protein B

Glycoprotein nonmetastatic melanoma protein B (GPNMB) is a type I transmembrane protein that was identified by the serial analysis of gene expression (SAGE) method as a potential GBM marker gene [123]. While normal brain samples expressed little or no GPNMB, 70% (35/50) and 66% (52/79) of GBM patient tumor samples were positive for the transcript and protein, respectively [124]. Survival analysis revealed that patients with relatively high *GPNMB* transcript levels, >3-fold over normal brain, as well as positive immunohistochemistry, had a significantly higher risk of death, making GPNMB an ideal target for the treatment of malignant glioma [124]. Human GPNMB protein is composed of 560 amino acids and is made up of three domains, a long ECD (464 amino acids), a single transmembrane region, and a short cytoplasmic domain (53 amino acids) [124]. An affinity-matured GPNMB-specific mutant scFv clone, F6V, was isolated from a human synthetic phage-display library [125]. The F6V scFv clone was fused to PE38 to generate the immunotoxin F6V-PE38. The F6V-PE38 exhibited significant cytotoxicity, IC_50_ = 8 pM, against GPNMB-expressing glioma cells, D293MG, D54MG, D245MG, and D212MG. Furthermore, F6V-PE38 delayed tumor growth over 17 days against subcutaneous malignant glioma xenograft D212MG [125].

### 3.7. High-Molecular-Weight Melanoma-Associated Antigen

The high-molecular-weight melanoma-associated antigen (HMW-MAA) consists of a 250 kDa N-linked glycoprotein and a proteoglycan component of >450 kDa [126]. The 250-kDa core glycoprotein encompasses a 2222 amino acid ECD, a 25 amino acid hydrophobic transmembrane region, and a 75 amino acid cytoplasmic tail [126]. The HMW-MAA expression has been demonstrated on malignant gliomas and is recognized by the mAb 9.2.27 [127]. The 9.2.27 mAb was chemically conjugated to the whole PE molecule to obtain 9.2.27-PE. Intratumoral treatment of established human glioma xenografts in nude rats with 9.2.27-PE IT extended the survival of these animals from 30% to 90% [128]. The HMW-MAA is also recognized by the mAb Mel-14 [129]. In an immunohistochemical analysis, 28 of 49 (57%) GBM patient specimens exhibited Mel-14 reactivity [130]. The ^131^I-labeled Mel-14 F(ab′)_2_ fragments were successfully tested in a Phase I clinical trial for neoplastic meningitis in a 46-year-old female who exhibited no radiographic evidence of disease, 4 years posttreatment [131, 132]. The Mel-14 scFv has been fused to PE38-KDEL to generate the immunotoxin Mel-14-PE38KDEL for targeted therapy of GBM expressing HMW-MAA [133].

### 3.8. EphA2

The Eph receptors consisting of 14 members are the largest family of tyrosine kinase receptors and are divided on the basis of sequence similarity and ligand affinity into A- and B-subclass. The ligands for Eph receptors, termed ephrins, are divided into the ephrin-A subclass (binds A-type receptor), which are glycosylphosphatidylinositol-linked proteins, and the ephrin-B subclass (binds B-type receptor), which are transmembrane proteins [134, 135]. Eph receptors and ephrins are both membrane bound, and hence, binding and activation of Eph and ephrins requires cell-cell interactions. Several biological functions, including vascular development, tissue-border formation, cell migration, axon guidance, and synaptic plasticity, have been attributed to Eph/ephrin interaction [135]. EphA2 is overexpressed in a number of solid tumors, including those of the breast, prostate, ovary, and pancreas [136]. The EphA2 receptor expression is also elevated in about 90% of GBM specimens and cell lines, while ephrinA1 is present at consistently low levels [137]. The EphA2 ligand ephrinA1 was chemically linked to PE38QQR. The cytotoxin ephrinA1-PE38QQR exhibited significant killing of GBM cells overexpressing the EphA2 receptor, with an average IC_50_ of ~10^−11 ^mol/L [136].

### 3.9. Urokinase-Type Plasminogen Activator Receptor

The urokinase-type plasminogen activator receptor (uPAR) is an important regulator of extracellular matrix (ECM) proteolysis, cell-ECM interactions, and cell signaling. uPAR is a member of the lymphocyte antigen 6 (Ly-6) superfamily of proteins, which is characterized by the Ly-6 and uPAR (Lu) domain. uPAR contains three Lu domains, designated D1–D3, connected by short linker regions [138]. The uPAR is associated with the external surface of the plasma membrane by a glycosyl phosphatidylinositol anchor. uPAR regulates the activity of the plasminogen activation system by binding to the amino-terminal growth factor domain of the serine protease urokinase-type plasminogen activator (uPA) [138]. uPAR is expressed in many human cancers, including bladder cancer, breast cancer, prostate cancer, colorectal cancer, gastric cancer, lung cancer, pancreatic cancer, glioblastoma, leukemia, and lymphoma [138]. Expression frequently indicates poor prognosis and in some cases is predictive of invasion and metastasis. A recombinant fusion protein, DTAT, containing the catalytic portion of DT for cell killing fused to the amino-terminal (AT) fragment of uPA, was tested for its effectiveness against uPAR-positive human GBM cells [139]. DTAT exhibited significant killing of uPAR-expressing GBM cells, U118MG and U87MG, *in vitro* and caused a statistically significant (*P* = 0.05) regression of small U118MG tumors in athymic nude mice, *in vivo* [139].

### 3.10. Bispecific Cytotoxins

To improve the efficacy of antiglioblastoma therapeutics, bispecific cytotoxins targeting two different antigens on tumor cells are being developed. A bispecific cytotoxin, DTEGF13, was constructed by fusing IL-13 to EGF and catalytically active truncated DT (DT_390_) in a single polypeptide chain [140]. Intratumoral injection of DTEGF13, but not monospecific DTEGF or DTIL13, significantly inhibited the growth of established U87 tumors in nude mice (*P* < 0.04) [140].

A second bispecific cytotoxin, EGFATFKDEL 7mut, was created by splicing EGF and amino-terminal fragment of uPAR (ATF) to a version of PE38KDEL in which the seven major immunodominant epitopes in PE38 were mutated to decrease toxin immunogenicity without a loss of catalytic activity [141]. Intracranial treatment of human GBM xenograft, U87, in athymic nude rats with EGFATFKDEL 7mut via CED led to significant reductions in tumor growth in two independent experiments (*P* < 0.01) [141]. Some rats remained tumor free over 130 days posttumor inoculation.

A bispecific cytotoxin DTAT13 was also synthesized in order to target simultaneously uPAR and IL-13R-expressing GBM cells. DTAT13 caused the regression of small tumors and was able to target both IL-13R and uPAR with less toxicity than DTAT or DTIL13 [142].

## 4. Future Directions

The therapeutic success of a tumor-targeting agent is influenced by several factors: selective killing of tumor cells in the absence of nonspecific toxicity, successful delivery of the immunotoxin to tumor cells, and finally, blocking the generation of anti-immunotoxin antibodies. Many of the molecular targets for immunotoxins are significantly overexpressed on the tumor cells but are also expressed on normal tissues, usually at a much lower level than on the tumor. Hence, it is essential to minimize the toxic effects of an immunotoxin owing to bystander cell death. The CED of macromolecules directly into the brain circumvents the blood-brain barrier, minimizes systemic exposure, with efficient intratumoral drug delivery and high local and peritumoral drug concentrations. The next concern is the successful conveyance of targeted toxins to tumor cells. Although CED provides the best volume of distribution of a locally administered drug, specific regions of the brain are naturally difficult to saturate completely with the infusate. Successful drug delivery will therefore depend on the proper placement of catheters. Advances in imaging and computational models will help to predict therapeutic agent distribution by CED and consequently can be used to optimize catheter positioning [143]. Moreover, imaging of therapeutic agent delivery during CED is essential to prove drug delivery to the intended target area [122] (Figure 1). A major disadvantage is that patients may develop neutralizing antibodies against toxins which prevent retreatment. To avoid the production of neutralizing antibodies against PE and to allow for more treatment cycles, a less immunogenic form of the PE38 has been generated by identifying and eliminating the B-cell epitopes on PE38 [144]. The new PE38 molecule has significantly less immunogenicity but still retains full cytotoxic and antitumor activities.

Brain tumors are highly heterogeneous, with multiple genetic alterations, and hence no tumor antigen is exclusively present on all tumor cells. Rather, it is a combination of antigens that defines the cells that make up the tumor population. With single-agent therapy, cancers tend to recur owing to overexpression of a different tumor antigen. Hence it is essential that a therapeutic regimen incorporate a cocktail of immunotherapeutic agents aimed at different cellular targets critical for brain tumor development, growth, and invasion. However, combination therapies involving multiple antibodies involve high development, manufacturing, and treatment costs. To overcome these difficulties, bispecific antibodies with different antigenic specificities are under development. As discussed above, the clinical utility and efficacy of bispecific antibody-based therapy is still in its infancy, with the majority of supporting evidence emerging from preclinical studies. The therapeutic success of a bispecific antibody in the clinical setting depends on the identification of the appropriate antigenic target combinations and the ability to produce sufficient quality and quantity of the therapeutic material.

 An alternative treatment strategy for brain tumor patients involves selective targeting of tumor cells by radioimmunotherapy (RIT). A potential advantage with RIT is the bystander effect of radiation, that is, depending on the path length of different isotopes it can kill adjacent tumor cells not expressing the target antigen. Several successful phase I and phase II trials in brain tumor patients with anti-tenascin mAbs BC-2, BC-4, and 81C6 labeled with the *β*-emitters, ^131^I and ^90^Y, and the *α*-emitter, ^211^At, demonstrated the therapeutic potential of RIT [145]. Further, a 46-year-old female with neoplastic meningitis was successfully treated in a phase I clinical trial with ^131^I-labeled Mel-14 F(ab′)_2_ targeting the HMW-MAA [132]. The therapeutic utility of mAbs targeting GAAs, EGFR, EGFRvIII, and podoplanin has been successfully demonstrated at the preclinical level [146, 147]. The RIT trials specified above involved locoregional administration of the radiolabeled mAbs. Optimization of RIT for future clinical trials involves the identification of an ideal radionucleotide and dosing regimen, along with improved intracerebral microinfusion by CED.

## 5. Conclusion

The present generation of targeted toxins has resulted from years of work by numerous investigators who have engineered these drugs to bind to receptors with reduced *in vivo* toxicity and immunogenicity. Immunotoxins exhibit a different mechanism of action than traditional chemotherapy and radiation therapy, hence might act synergistically with these forms of treatment without subsequent cumulative side effects. Further progress and improved clinical response depends on the identification of new antigenic targets on tumors and on administering a combination of immunotoxins or bispecific immunotoxins that target different tumor antigens.

## Figures and Tables

**Table 1:** Current listing of immunotoxin trials for brain tumor therapy.

Antigen targeted	Immunotoxin characteristics	Clinical trial	Tumor treated	Outcome	References
TR: Tf-CRM107	A conjugate of human TR and full-length DT with two point mutations in the B chain with reduced toxin binding (CRM107)	Phase I/II	44 patients (recurrent GBM or AA)	Median survival 37 weeks; 5/34 CR; 7/34 PR	[38, 39]
TR: 454A12-rRA	A conjugate of a mAb (454A12) to the human TR and rRA	Phase I	Eight leptomeningeal neoplasia patients	Tumor progression	[40]
IL-4R: IL-4(38-37)-PE38KDEL (NBI-3001)	A circularly permuted recombinant IL-4 fused to PE	Phase I/II	31 patients (25 GBM and 6 AA)	Median survival 8.2 months; 6 month survival 52%	[41–44]
IL-13R: IL-13-PE38QQR (Cintredekin Besudotox)	A chimeric toxin composed of hIL-13 and a mutated version of PE	Phase I/II/III	Phase II: 51 patients (46 GBM, 3 AA, 1 anaplastic oligoastrocytoma, 1 AO; Phase III: 296 patients (276 GBM confirmed)	Phase II: median survival 45.9 weeks Phase III: IL13-PE38QQR compared to GW: Median survival 36.4 weeks for the IL13-PE38QQR group and 35.3 weeks for the GW group	[45, 46]
EGFR: TP-38 (IVAX)	A chimeric toxin composed of EGFR ligand TGF-*α* and PE	Phase I	Twenty patients [17 GBM, 1 GSC, 1 metastatic spindle cell sarcoma, 1 AO]	Median survival 28 weeks	[47–49]
Mutant EGFRvIII: MR1-1-PE38KDEL	An affinity-matured scFv specific for EGFRvIII, MR1-1 fused to PE	Phase I	Ongoing		

## Abbreviations

AA: Anaplastic astrocytoma
AO: Anaplastic oligodendroglioma
CR: Complete response
DT: Diphtheria toxin
EGFRvIII: Epidermal growth factor receptor variant III
GBM: Glioblastoma multiforme
GSC: Gliosarcoma
GW: Gliadel wafer
IL-4R: Interleukin-4 receptor
IL-13R: Interleukin-13 receptor
mAb: Monoclonal antibody
PE: *Pseudomonas* exotoxin A
PR: Partial response
rRA: Recombinant ricin A chain
scFv: Single chain variable region antibody fragment
TGF-*α*: Transforming growth factor *α*
TR: Transferrin receptor.
